# Clinical spectrum and uncommon features of McCune-Albright syndrome in children: a cohort study from a National Referral Center

**DOI:** 10.3389/fendo.2025.1531765

**Published:** 2025-02-26

**Authors:** Solène Bergignat, Roland Chapurlat, Marc Nicolino, Kevin Perge

**Affiliations:** ^1^ Hospices Civils de Lyon, Hôpital Femme Mère Enfant, Service d’Endocrinologie Pédiatrique et Pédiatrie Générale, Bron, France; ^2^ Faculté de Médecine Lyon-Est, Université Claude Bernard, Lyon, Lyon, France; ^3^ Hospices Civils de Lyon, Hôpital Edouard-Herriot, Service de rhumatologie, Lyon, France; ^4^ Centre National de Référence de la Dysplasie Fibreuse des Os, Hôpital E Herriot, Lyon, France

**Keywords:** McCune-Albright syndrome, *GNAS*, precocious puberty, acromegaly, thyroid carcinoma, Cushing syndrome, hepatocellular adenoma, fibrous dysplasia

## Abstract

**Introduction:**

McCune-Albright syndrome (MAS) is a rare disease caused by somatic gain-of-function variants in the *GNAS* gene that lead to constitutive activation of the G protein alpha subunit (Gsα). Pathologic consequences can involve several tissues. Fibrous dysplasia (FD), café-au-lait skin macules and hyperfunctioning endocrinopathies are classic manifestations. However, the phenotypic spectrum of MAS is considerably wider and more complex.

**Methods:**

We performed a pediatric retrospective study from our National Referral Center between 2007 and 2021 to describe the clinical spectrum of MAS in children, with a focus on unusual or severe manifestations.

**Results and discussion:**

A total of 33 children were included. Peripheral precocious puberty was the most frequent endocrinopathy, affecting 84,6% of girls and was the presenting feature for 57,6% of them. Thyroid involvement was also common, consisting in morphological abnormalities with or without slight hyperthyroidism. Thyroid nodules were typically benign, but one patient presented a follicular thyroid carcinoma. Cushing syndrome typically occurs in the neonatal period, but we observed an unusual case of hypercortisolism revealed in early infancy. FD was very common and manifested along a wide range of severity, from monostotic and asymptomatic lesion to polyostotic FD with pain, fractures, and compressive optic neuropathy. We described a locally aggressive FD involving sphenoid and maxillary bones which leaded a young female patient to death. Finally, we reported hepatic disorders, including a case of hepatocellular adenoma. In conclusion, MAS is a multisystemic disorder, with a variable combination of symptoms, and a broad range of severity. These uncommon abnormalities mostly occurred in patients with significant involvement of multiple other tissues.

## 1 Introduction

McCune-Albright syndrome (MAS; OMIM#: 174800) is a rare disease with estimated prevalence between 1/100 000 (1). It is caused by somatic gain-of-function variants in the *GNAS* gene, which encodes the α-subunit of the Gs protein (Gαs), leading to constitutive Gαs activation (2–4). Pathologic consequences of the over-activation of this signaling pathway can involve several tissues (5). Fibrous dysplasia (FD), café-au-lait skin macules, and hyperfunctioning endocrinopathies are the classic manifestations of MAS. However, the phenotypic spectrum of MAS is considerably wider and more complex. The new diagnostic criteria for MAS are the combination of FD and one or more extra-skeletal features, OR the presence of two or more extra-skeletal features, FD not always being associated as previously described (5). Thus, the clinical presentation of patients is highly variable and will depend on the unique pattern of affected cells (4). The aim of this study was to describe the clinical spectrum of MAS in children and to focus on unusual or severe manifestations.

## 2 Methods

We performed a retrospective monocentric study on children followed in the National Referral Center of Fibrous Dysplasia in the Hospices Civils de Lyon (France). All the children (aged 0 to 18 years old) with a diagnosis of MAS followed in this center from 2007 to 2021 were included. The diagnosis of MAS was clinical, based on the combination of FD and one or more extra skeletal feature, or the presence of two or more extra skeletal features. Extra skeletal features included café au lait skin macules, gonadotropin-independent precocious puberty, testicular lesions with or without associated precocious puberty, thyroid lesions with or without hyperthyroidism, growth hormone excess and neonatal Cushing syndrome. Data regarding demographic parameters, clinical and biological phenotypes, and genotype (detection of *GNAS* p.R201H and p.R201C variants using droplet digital PCR in blood sample or, in some cases, in other tissues [thyroid, bone … ]) were extracted from the medical records of each case. Two authors (SB and KP) reviewed each case. In accordance with current French regulations, parents were informed of the study by postal mail, and were given the possibility to refuse participation in the study.

## 3 Results

### 3.1 Main characteristics of patients

**Figure 1:**
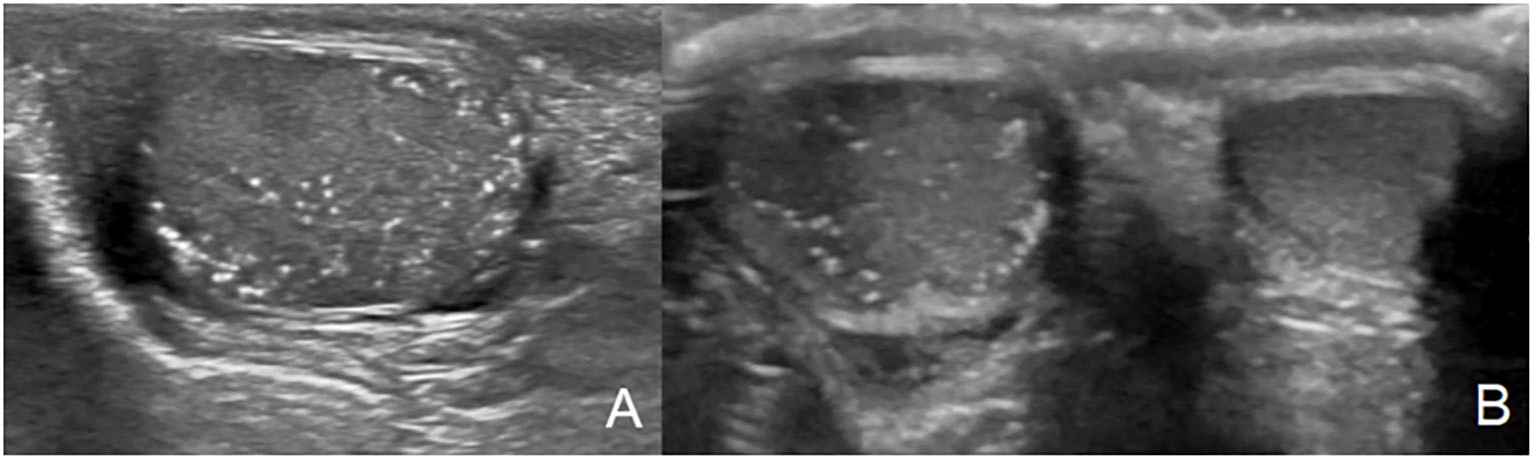
Testicular microlithiasis characterized by multiple small, same-sized echogenic non-shadowing foci **(A, B)**.

**Figure 2:**
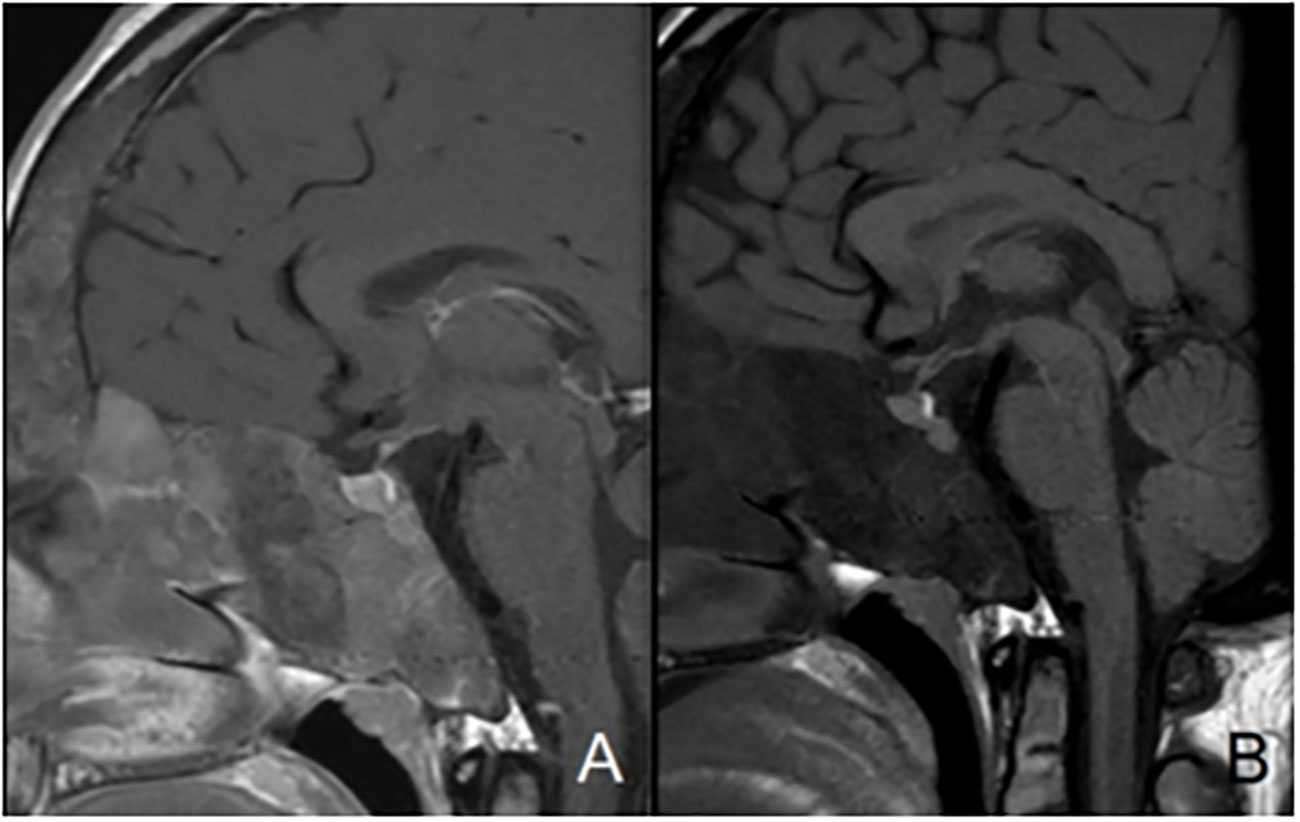
Mid-sagittal T1-weighted images with **(A)** and without **(B)** contrast-enhancement showing normal posterior pituitary gland and pituitary stalk and increased anterior pituitary hyperplasia in a patient with sphenoidal and clival bone dysplasia.

**Figure 3:**
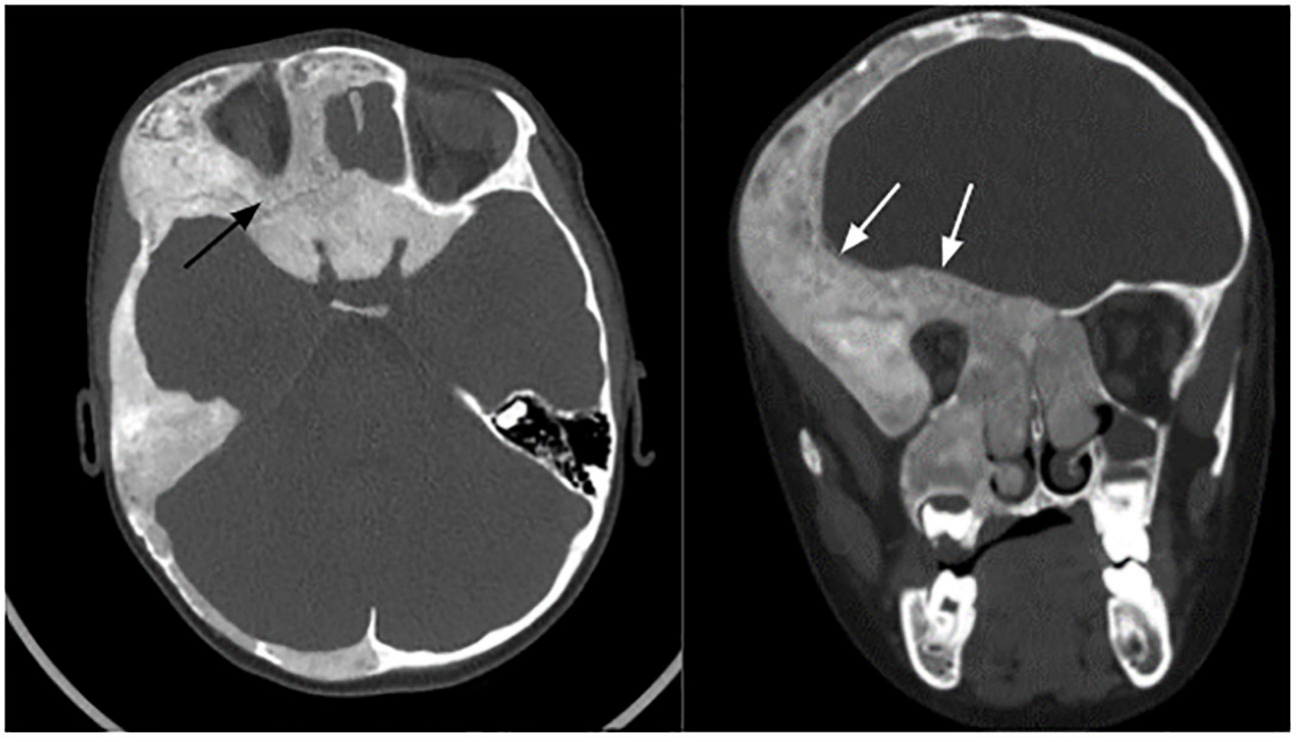
Cranio-facial fibrous dysplasia (white arrows) with narrowing of optic canals (black arrows).

**Figure 4:**
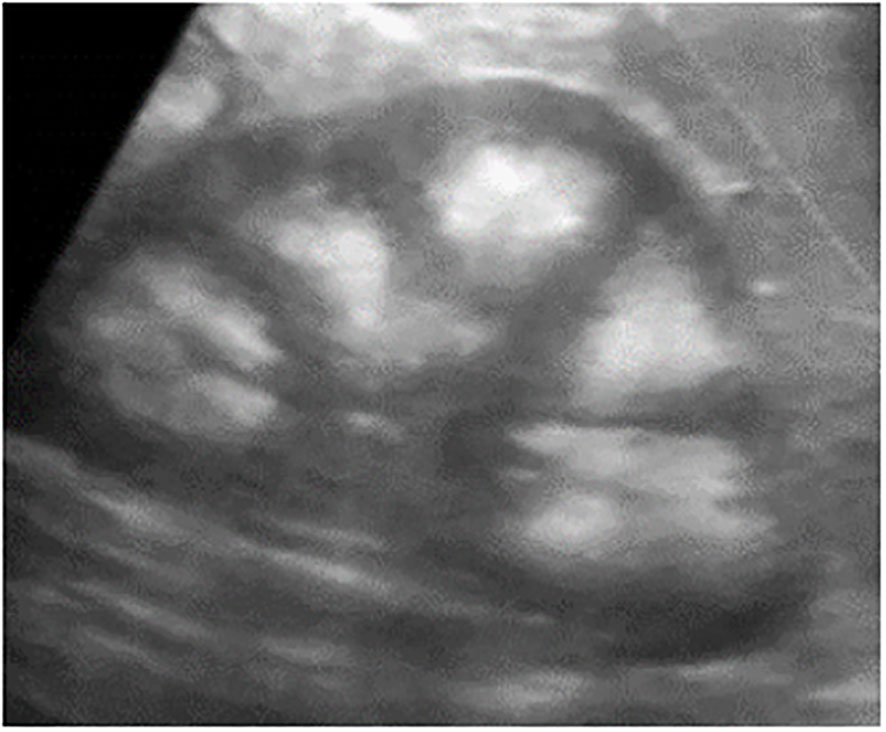
Grade 3 nephrocalcinosis in a 5 years child who had a severe neonatal Cushing syndrome.

A total of 33 children with MAS were included. The major findings of our cohort are summarized in **Tables 1**, **2**. The sex ratio male/female was 0,27 and the median age at diagnosis was 4.5 years (range: 0.1-9.5). The classic triad of FD, café-au-lait spots and precocious puberty was identified in 16 patients (48,4%). Skins lesions with classical description (café au lait spots with jagged, irregular borders in « Coast of Maine ») were observed in 90% of patients. Peripheral precocious puberty was the most frequent endocrinopathy in girls, affecting 22/26 (84,6%) of them, at a median age of 4.5 years (range: 1-6.5). It was the presenting feature of MAS for 57,6% of them. Fifty-five percent of these girls progressed to central precocious puberty. We did not identify case of ovarian torsion. Precocious puberty was less frequent in boys, concerning 42,9% of them. Additional findings of MAS such as testicular microlithiasis (**Figure 1**) and asymmetric volume of the testes were observed in 1 and 2 patients respectively. We found thyroid morphological abnormalities in 36% of patients (9/25) on thyroid ultrasound, with nodular or multinodular thyroid. Nodules were typically benign micronodules (< 1 cm), and the size of the thyroid was normal in 77,8% of cases. However, we identified one case of thyroid carcinoma in a girl, case previously described in the literature (see uncommon features of MAS) (6). Twelve percent (4/33) of patients developed slight hyperthyroidism, subclinical in most cases. Fifteen percent of patients experienced growth hormone (GH)-excess (age range of GH-excess diagnosis: 2.7-17.6 years). All these patients presented with skull base FD (**Figure 2**). In one case, GH excess probably participated to FD expansion (**Figure 3**). Hypercortisolism concerned two patients. One of them experienced severe neonatal Cushing syndrome at one-month-old and underwent bilateral adrenalectomy. Hypertrophic cardiomyopathy, abnormalities of liver function test and hyperglycemia resolved but nephrocalcinosis persisted (**Figure 4**). We identified one case of atypical hypercortisolism diagnosed late at age four, case previously described in the literature (see uncommon features of MAS) (7). FD concerned 81,8% of patients, with a median age at diagnosis of four years old (range: 0.1-13.5 years). FD was polyostotic in 92,5% of patients and involved craniofacial bones in 85% of cases. Compressive optic neuropathy concerned 9% of patients. We observed rare other FD complications: bone cysts, locally aggressive fibrous dysplasia (case previously described in the literature), Chiari I malformation (see uncommon features of MAS) (8).

**Table 1:** Prevalence of major findings in our cohort of patients with MAS.

Clinical feature	Number of patients ^1^
Café-au-lait skin macules	30 (90,9%)
Fibrous dysplasia of bone	27 (81,8%)
Precocious puberty (girls)	22 (84,6%)
Precocious puberty (boys)	3 (42,9%)
Hyperthyroidism or thyroid nodules	9 (27,3%)
GH-excess	5 (15,2%)
Hypercortisolism	2 (6,1%)

**^1^** n = 33, 26 girls, 7 boys.

**Table 2:** Demographic, clinical and genetic characteristics of our cohort of 33 patients with MAS.

Patient	Sex	Age at diagnosis (years)	Main clinical features	Presence of *GNAS* mosaic variant	Age at last evaluation (years)
1	F	9,5	FD, TH, CAL	NA	15,7
2	F	4,9	FD, PP, GH, PRL, CAL	NA	11,1
3	F	2,4	FD, PP, TH, GH, PRL, CAL	+ (blood)	11,3
4	F	0,1	FD, PP, CS, TH, CAL, HEP	+ (adrenals, conjunctiva)	5,6
5	F	0,8	FD, PP, TH, CAL	+ (blood)	15,5
6	F	3	PP, CAL	NA	21
7	M	3,6	FD, CAL	NA	8,5
8	M	5,9	FD, PP, TH, CAL	NA	13,5
9	F	6	FD, PP, TH, PRL, CAL	+ (blood, thyroid)	19
10	F	1,5	FD, PP, CAL	-(blood)	9,8
11	F	5,4	FD, PP, GH, PRL, CAL, HEP	NA	11,8
12	F	3,3	FD, PP	NA	5,5
13	F	0,3	FD, PP, CS, TH, CAL, HEP	+(blood, liver)	19,8
14	F	5,5	PP, CAL	NA	13
15	F	6,5	FD, PP, CAL	+ (blood)	12,3
16	F	4,5	PP, CAL	+ (blood)	11,6
17	M	NA	FD, PP, TH, CAL	NA	10,7
18	F	2,6	FD, PP	- (blood)	17,8
19	F	5	PP, CAL	NA	8,7
20	F	5	FD, PP, CAL	+ (blood)	13,4
21	M	2,2	FD, CAL	NA	6,1
22	F	1,8	FD, PP	+ (blood)	16
23	F	8,7	FD, PP, CAL	NA	15,3
24	F	5,1	FD, PP, CAL	NA	13,3
25	F	4,5	PP, CAL	NA	8,3
26	F	7,8	FD, CAL	NA	11,3
27	F	4	FD, PP, TH, CAL	- (blood)	15
28	M	7,6	FD, CAL	+ (blood)	10,1
29	M	4	FD, CAL	NA	24
30	M	0,9	FD, PP, GH, TH, PRL, CAL	+ (blood)	19,6 (death)
31	F	6,3	PP, CAL	NA	13
32	F	8,1	FD, CAL	- (blood)	11,3
33	F	1,4	FD, CAL	+ (bone) -(blood)	5,1 (death)

CAL, café-au-lait skin macules; CS, Cushing syndrome; FD, fibrous dysplasia; HEP, hepatic involvement; NA, not available; PP, precocious puberty; PRL, hyperprolactinemia; TH, thyroid abnormalities.

Finally, we observed two other uncommon features of MAS: hepatobiliary manifestations (neonatal hepatic cytolysis and cholestasis) and an exceptional case of infantile glaucoma (see uncommon features of MAS).

### 3.2 Uncommon features of MAS

#### 3.2.1 Rare case of thyroid carcinoma

**Figure 5:**
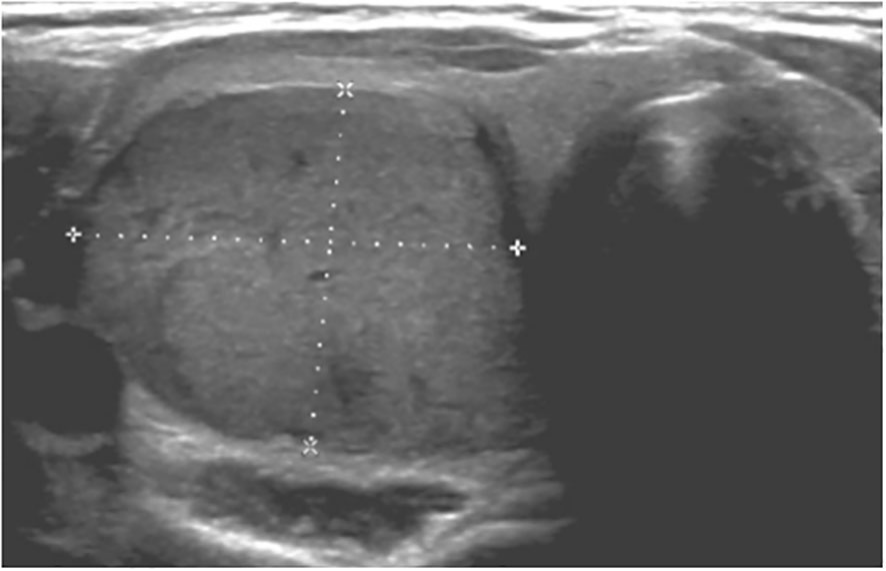
Thyroid carcinoma. Hypervascularized nodule of 25 × 20 × 30 mm in the right lobe.

The patient was diagnosed at the age of six with MAS based upon identification of the association of typical café-au-lait spots and peripheral precocious puberty (6). She also presented severe polyostotic FD, predominant on the right side of the skeleton and involving the skull base, and renal phosphate wasting. She underwent preventive osteotomy on the right femoral neck and received bisphosphonate therapy. At age 17, a systematic follow-up evaluation was realized, including thyroid ultrasound and blood analysis. Ultrasound revealed one voluminous and hypervascularized nodule of 25 × 20 × 30 mm in the right lobe and two nodules of eight and seven mm in the left lobe All these nodules were classified EU-TIRADS (European Thyroïd Imaging-Reporting and Data System: risk score of malignancy) 3 (low risk of malignancy) (**Figure 5**). Biologically, she presented suppressed TSH, less than 0,01 mUI/ml (normal: 0,4 – 3,1), slight elevation of T3 at 5,2 and 5,6 pmol/L (normal: 2,9 – 4,9) and T4 levels at 11,2 and 14,5 pmol/L (normal: 12 – 22). She had no symptoms of thyrotoxicosis. A technetium-99 m pertechnetate thyroid scintigraphy scan showed a strong hyperfixation at the location of the nodule. A right hemithyroidectomy was performed and the pathology revealed a follicular thyroid neoplasm. The R201C *GNAS* pathogenic variant was detected in neoplastic thyroid cells. No other mutation (including BRAF and N RAS) was detected. This thyroid carcinoma developed in a context of emerging GH-excess for which an intramuscular preparation of long-acting sandostatin was introduced two months later.

#### 3.2.2 Rare case of Cushing syndrome

Diagnosis of MAS was made at age six months, in the presence of diffuse hyperpigmented spots on the right buttock and abdomen, severe FD and *GNAS* gene variant in peripheral leucocytes (7). A growth deceleration started at age eight months. Investigation of this growth retardation revealed abnormally high levels of dehydroepiandrosterone sulfate (DHAS) and a loss of cortisol diurnal variation (**Table 3**).

**Table 3:** Evolution of adrenal hormones rates in our patient from 2 to 17 years old.

Years	Ketoconazole								
	2	4.2	5	5.5	7.8	10.8	11.5	14.2	17.8
Serum cortisol levels (nmol/L)									
4 am	NA	311	232	118	187	183	239	223	300
8 am	386	355	415	118	266	369	302	256	440
12 am	365	358	954	157	200	268	320	188	NA
4 pm	NA	360	1245	145	228	221	162	203	NA
8 pm	329	308	1181	114	279	138	106	121	207
12 pm	NA	275	270	78	185	125	102	102	129
Serum ACTH levels (ng/ml)									
4 am	NA	NA	2	7	<5	4	7	10	<3
8 am	9	2	4	7	6	9	<3	4	4
12 am	6	2	3	8	<5	5	5	<3	NA
4 pm	NA	2	3	8	5	3	<3	3	NA
8 pm	10	2	3	8	<5	<3	<3	<3	<3
12 pm	NA	1	2	1	6	<3	<3	<3	<3
**Urines free cortisol levels (nmol/24h)**	66	115	2330	23	NA	NA	68	82	NA
**Serum DHEAS levels (nmol/L)**	1944	1930	4676	1659	NA	1682	1617	1332	NA

Normal ranges: DHEAS (nmol/L), 1 - 5 years: 59 +/- 41, 6-9 years: 320 +/- 250, P1: 1800 +/- 620, P2: 1400 +/- 700, P3: 1850 +/- 1300, P4: 2425 +/- 1330, P5: 3120 +/- 1310; urines free cortisol, 30 à 60 nmol/24h in young children, 10 – 105 nmol/24h in adult women.

N.A., not available.

She was mildly obese (BMI: IOTF 33), but she had no other cushingoid features. At age 4 years, the diagnosis of adrenocorticotropic hormone (ACTH)-independent hypercortisolism was confirmed, with obviously pathological serum and urine cortisol levels, and concomitant low ACTH levels. Ultrasound and magnetic resonance imaging (MRI) revealed slightly enlarged adrenal glands but no nodules. Cerebral MRI did not show pituitary adenoma. A few months later, cortisol levels strongly increased, and a medical treatment by oral ketoconazole was introduced. A good control of hypercortisolism was obtained with Ketoconazole, which was stopped at age 11 years. No recurrence was observed after eight years of follow-up.

#### 3.2.3 Complications of fibrous dysplasia of bone

##### 3.2.3.1 Bone cysts

**Figure 6:**
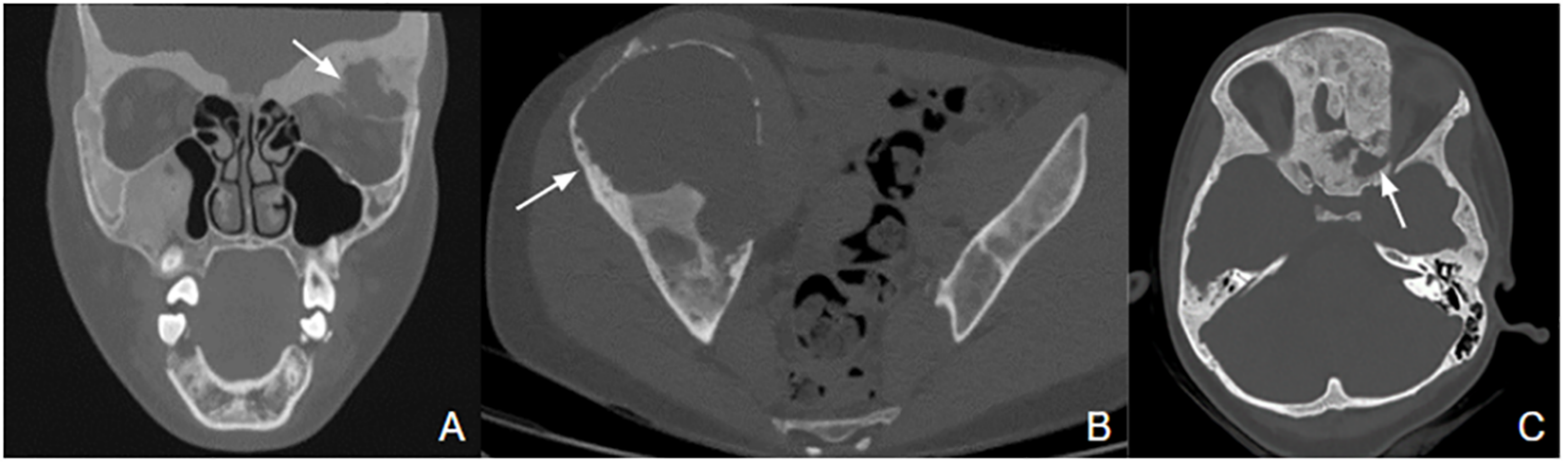
FD with bone cysts. **(A)** Mixed solid and cystic formation (white arrow) in the left orbit, responsible for a mass effect on ocular muscles and eyeball with exophthalmos. **(B)** Giant cystic lesion (white arrow) with a diameter of 15 cm in the right iliac wing, causing pain and limp. **(C)** Cyst of 18x26x18 mm (white arrow) in left spheno-orbital fibrous dysplasia, causing narrowing of the superior orbital fissure and compression of the left optic nerve.

Three patients developed cystic formation in FD (**Figure 6**). Among these three patients, a 11-year female patient suddenly lost vision of one eye because of the development of an intraosseous cyst. A few days before her vision loss, she presented left peri-ocular headache. A medical treatment with steroids at 1 mg/kg for seven days, then a surgical decompression of the optic nerve was attempted but did not improve the symptoms.

##### 3.2.3.2 Locally aggressive fibrous dysplasia

A large mass involving the sphenoid and the right maxillary bones developed in a 17-month female patient (8). Diagnosis of FD was made by histological examination of the mass. She also presented café-au-lait skin spots, but no endocrinopathy was detected. The evolution of this mass was extremely aggressive, with a recurrence shortly after the first surgical removal. Because of the unusual evolution, a second biopsy was performed, and still showed FD lesions without sign of malignant transformation. Genetics analysis showed the presence of the R201H *GNAS* pathogenic variant in cells of the osseous mass but not in peripheral leukocytes. A chemotherapy was attempted to decrease the tumoral vascularization at age 4.8 years for 2 months, but was not effective, and the patient died at five years.

##### 3.2.3.3 Chiari I malformation

**Figure 7:**
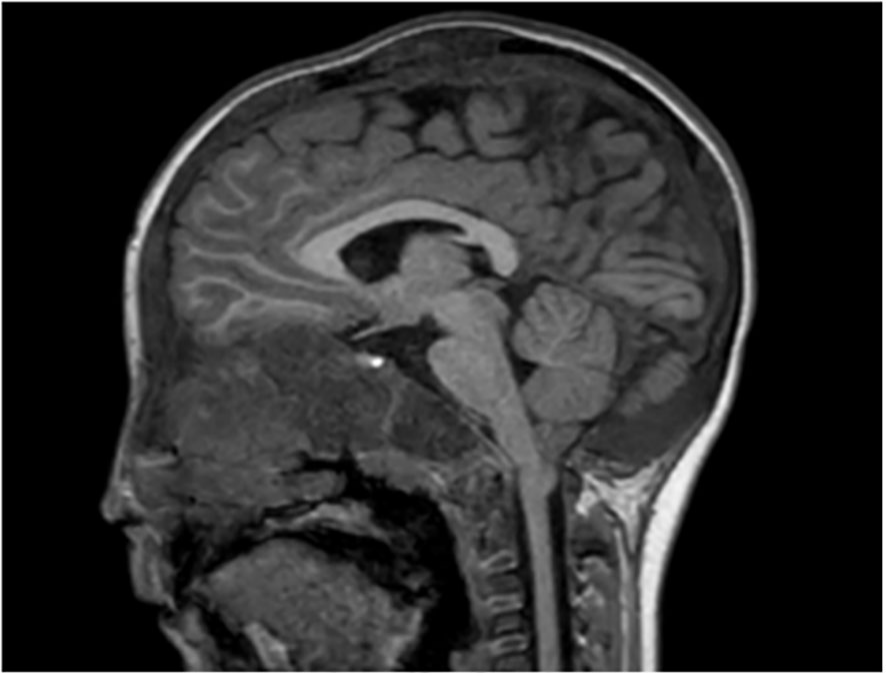
Asymptomatic Chari I malformation in a patient with MAS. Cerebellar tonsils ptosis of 5 mm under foramen magnum plane.

We incidentally identified Chiari I malformation in a patient with severe DF involving the skull base (**Figure 7**). The patient did not present any symptom linked to this malformation.

#### 3.2.4 Hepatobiliary manifestations

In our series, we observed two cases of neonatal hepatic dysfunction. One case was included in a context of neonatal Cushing syndrome. Regarding the second patient, the link with MAS isn’t clearly established, but the etiological assessment was negative. The patient presented hepatic cytolysis and cholestasis with normal Gamma Glutamyl Transferase level, and the biopsy revealed giant cell hepatitis. The usual etiological work-up (viral, autoimmune, metabolic) was performed.

**Figure 8:**
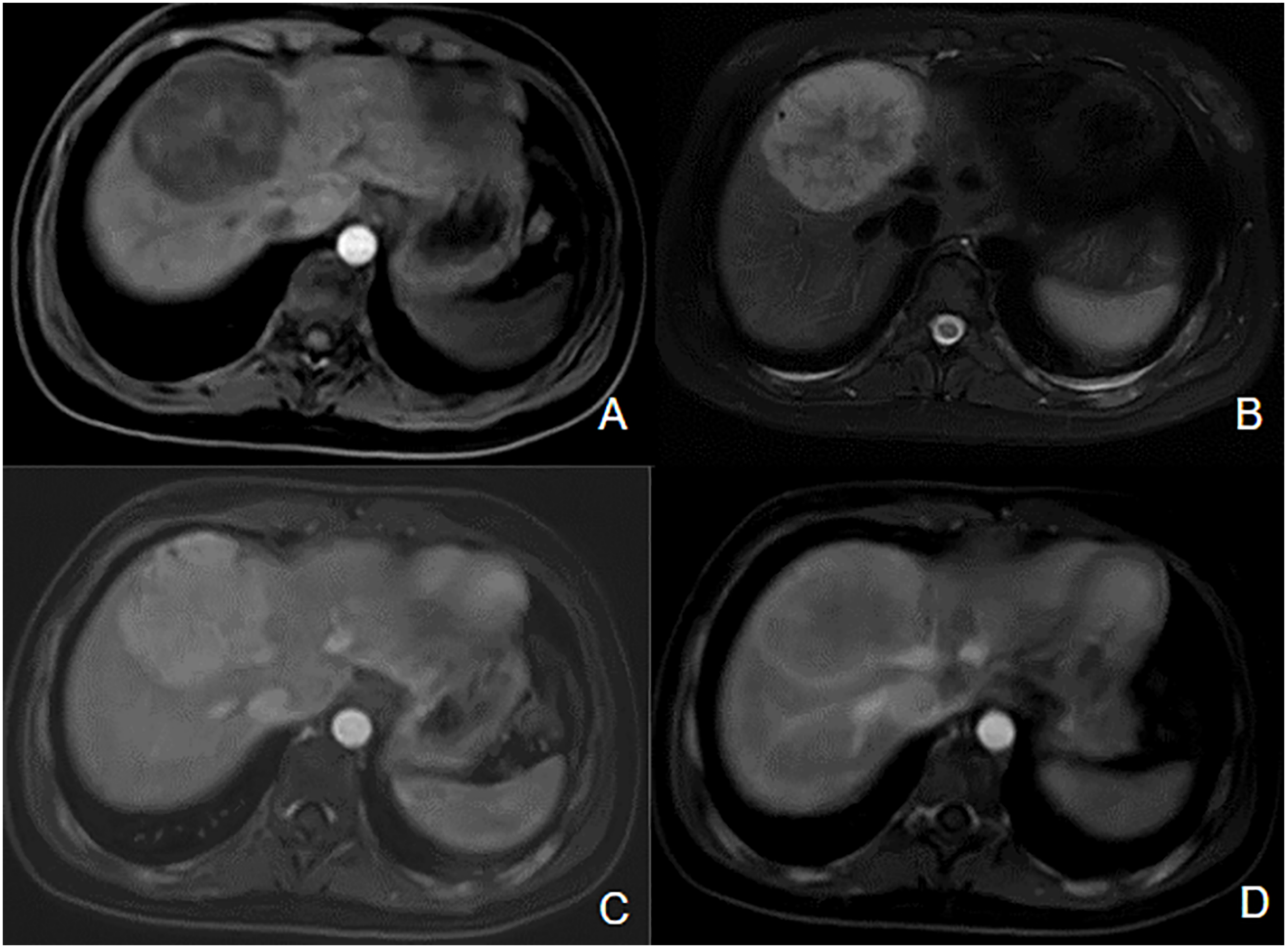
Inflammatory hepatocellular adenoma in a patient with MAS. Hypointense mass located on the junction of segments VIII and IV of the liver on axial T1-wieghted image **(A)**, hyperintense on T2-weighted image **(B)**. We can observe enhancement on the T1-postcontrast arterial phase image **(C)**, persistent during the portal phase **(D)**.

We also observed a hepatocellular adenoma in a patient with severe MAS. The liver lesion was detected at age 17 years on a follow-up ultrasound, and initially measured 42 mm (**Figure 8**). The patient was asymptomatic, and she did not use a contraceptive pill. A resection was realized 18 months later because of its rapid growth to 75 mm and the inherent risk of bleeding. Anapathological examination confirmed hepatocellular adenoma without degeneration and the R201C pathogenic variant was detected in resected tissue.

#### 3.2.5 Infantile glaucoma

Our patient presented a very severe phenotype of MAS, revealed by a neonatal Cushing syndrome. She was managed with bilateral adrenalectomy. She also had precocious puberty, severe FD, café-au-lait spots, transient hyperthyroidism, and severe developmental delay. At age five years, we observed a frank buphthalmia in the left eye, associated with diffuse corneal edema, important scleromalacia and enlargement of the limbus. Diagnosis of infantile glaucoma was supported by increased intraocular pressure and cup to disc ratio. A medical treatment based on eye drops was attempted but did not improve the symptoms. A trabeculectomy was then decided. During the surgery, ocular biopsies were realized, and R201H *GNAS* pathogenic variant was detected in conjunctiva and iris samples.

## 4 Discussion

To our knowledge, our study constitutes one of the largest pediatric series of patients with MAS.

### 4.1 Cutaneous lesions

Skins lesions are the most frequent feature of MAS, affecting 90% of patients in our cohort. The absence of these skin lesions should not rule out the diagnosis of MAS. Café au lait spots usually appear in the neonatal period and are characterized by jagged, irregular borders in « Coast of Maine » (9, 10). Their distribution typically follows the midline of the body (10). Surprisingly, despite being present from birth, skin involvement is not the most common telltale sign of MAS. Thus, a wider awareness of this characteristic lesions is necessary and the presence of them should encourage the clinician to discuss the diagnosis of MAS and an assessment and, if necessary, follow-up of the patient in a reference center (10).

### 4.2 Gonadal manifestations

Our study reminds us that gonadal involvement is the most common endocrine involvement in MAS. Peripheral precocious puberty is even the first sign of MAS in most cases.

#### 4.2.1 Gonadal manifestations in girls

Peripheral precocious puberty is linked to intermittent formation of autonomous ovarian cysts, which are the morphological manifestation of the hyperactivation of follicular cells (11, 12). The age of onset of puberty is in the majority of cases before the age of six, as has been described in others cohorts (9, 10). As precocious puberty is often the first manifestation of MAS, tumoral hypothesis can be raised and lead to unnecessary oophorectomy. Nabhan et al. reported a series of nine girls who presented sudden vaginal bleeding associated with an ovarian mass: of these, four patients underwent a salpingo-oophorectomy before the diagnosis of MAS was made (13). Moreover, despite the presence of large ovarian cysts, the risk of ovarian torsion remains low (14). We identified no case of ovarian torsion in our cohort. Our cohort illustrated the risk of progression to central precocious puberty as any peripheral precocious puberty, a risk that should encourage close clinical and biological monitoring in order to add, if necessary, treatment with gonadotropin-releasing hormone analogs. Finally, it would be interesting to follow up our cohort in order to obtain data on fertility, data hitherto limited in MAS. A recent study in the USA showed that fertility is impaired in women with MAS (43% prevalence of infertility, compared with 10.9% in the national average), but the possibility of spontaneous conception remains (14).

#### 4.2.2 Gonadal involvement in boys

Peripheral precocious puberty is much less frequent in boys, affecting 0-40% of them, with heterogeneous data (15–17). Results from cohort were comparable with 43% of boys affected. Histologic findings include Leydig and Sertoli cell hyperplasia (16). Macroorchidism without precocious puberty may also be observed if MAS is restricted to Sertoli cells (11). While gonadal involvement may be clinically silent, lesions are very common on ultrasound. Indeed, in a cohort of 54 males, Boyce et al. observed 81% of ultrasound testicular abnormalities, including hypoechoic lesions, hyperechoic lesions, microlithiasis, or diffuse heterogeneity (16). Finally, testicular microlithiasis described in one of our patients has been reported in 24,1% to 62,5% of males in two studies including boys and men (16, 18). The clinical relevance and evolution of these microlithiases are poorly known. To the best of our knowledge, only one case of testicular cancer (embryonal cell tumor) has been reported in context of MAS, in a 28-yr-old man (17). Gonadal abnormalities in men may affect fertility but data in men is very limited (15).

### 4.3 Thyroid abnormalities

Thyreopathy is the second most common endocrinopathy in children and adolescents with MAS (19). Possible abnormalities include nodular, multinodular thyroid, and hyperthyroidism, subclinical in most cases (19–21). The rate of ultrasonographic lesions reported in the literature range from 14% to 54% (19–22). Biochemically, *GNAS* variants result in constitutive 5′-deiodinase activity, resulting in increased conversion of T4 to T3 (22). Our study as our previous report illustrates the risk of progression of thyroid nodules to thyroid malignancies in MAS (6). Association of MAS with thyroid cancer seems rare. Some other cases of thyroid malignancies with different histological profiles are reported (23–25). Another case of lipid-rich follicular carcinoma of the thyroid was described in a 41-year-old woman (26). Gsp, the constitutively active forms of GNAS generated by hotspot variants, is emerging as an oncogene acting in multifactorial transformation processes in low-grade or benign neoplasia (27). Indeed, activating *GNAS* variants are associated with various sporadic endocrine and non - endocrine tumors as intraductal papillary mucinous neoplasms, GH-secreting adenoma, pituitary toxic adenoma (27). According to Yoshimo and al, *GNAS* variants at codons 201 or 227 may play a role in the pathogenesis of a small population of papillary thyroid carcinomas (28). Finally, we can also wonder if GH-excess participated to the development of the thyroid carcinoma in our patient. Another case of thyroid malignancy previously mentioned was associated to poorly controlled acromegaly (25). Wonliski et al. reported in a meta-analysis an increased risk of thyroid nodules and thyroid cancer in acromegalic patients than in general population (29). All of these data should encourage the clinician to set up annual clinical, biological and ultrasound monitoring of thyroid in patients presenting MAS.

### 4.4 Pituitary manifestations

Pituitary tumors described in MAS are almost exclusively GH and/or prolactin-secreting adenomas. As MAS-associated GH excess occurs at a mean age of 18-24 years, its prevalence is higher in cohorts with adults: 19-25% (30–32). GNAS variant can be responsible for somatotroph hyperplasia involving the entire pituitary gland, with or without development of a somatotroph adenoma (33). Pituitary adenoma is identified in just over half of cases, which is significantly lower than in patients with classic acromegaly (33). GH excess is associated with hyperprolactinemia in 46%-81,4% of cases (25). In our series as in the literature, skull base FD appears as a consistent finding in patients with MAS-associated GH excess (30, 34). Moreover, GH excess could participate to FD expansion and other manifestations, as seen previously. Indeed, acromegaly is associated with an increased risk of optic neuropathy (30, 34, 35), with a decreased risk in case of early treatment (34). Thus, we suggest annual biological screening for acromegaly and hyperprolactinemia in patients with MAS, especially those with skull base FD.

### 4.5 Adrenal involvement

Hypercortisolism is a rare feature of MAS, which classically presents during the first year of life (36, 37). The median age at diagnosis is three months (37–39), and its occurrence beyond a few months of life is exceptional. It is one of the most severe features of MAS, lethal in 20% of cases (36, 39). Cardiac and liver involvement can be associated with Cushing syndrome and constitute poor prognosis markers (36). Hypercalciuria and nephrocalcinosis are also seen in 30% to 60% of cases (36, 39). MAS-associated Cushing syndrome has been described in neonatal period and is uncommon after the first year of life (36, 37). It classically corresponds to diffuse and nodular cortical hyperplasia associated to multifocal cortical atrophy, constituting a bimorphic cortical pathology. Its occurrence in neonatal period parallels the involution of the fetal adrenal gland and may suggest a differential effect of the gsp variant on the fetal adrenal (10, 37). According to Breault et al, protein subunit αs is expressed in both definitive zone and fetal zone, but less intensely in the definitive cells than the fetal cells (40).

We here report an unusual case of hypercortisolism revealed after neonatal period, in early infancy, in addition with two cases previously reported at three and 17 years old (7, 37, 41). Thus, these rare cases should encourage us to remain cautious and to carry out biological assays (urinary free cortisol, 24-hour cortisol cycle, dexamethasone braking test) at the slightest suspicion of Cushing syndrome.

### 4.6 Fibrous dysplasia of bone

FD can manifest along a wide spectrum: from an isolated, asymptomatic monostotic lesion to severe, disabling polyostotic disease (9). Most skeletal lesions and the associated functional disability (pain, limp, fractures, loss of mobility) occur within the first decade of life (42). The maximal fracture rate occurs between the ages of six and ten years (43). The proximal femur is commonly affected (44). FD lesions should be monitored, and preventive surgery should be considered in case of threatening osteolytic lesion. Cranio-facial FD can cause pain, facial deformities, exophthalmos, and compression of adjacent structures as optic nerves or external auditory canals (9). As described in our cohort, optic nerve encasement is a frequent feature in patients with craniofacial FD (45). However, most patients with encased optic nerves do not exhibit symptoms of optic neuropathy (35, 45, 46). The mechanism of compression can be slowly progressive, or more rarely acute, caused by aneurysmal bone cyst as reported in one case of our cohort. Thus, unusual headache in a patient with craniofacial FD should raise the alarm and lead to the search for an acute complication. Our report of locally aggressive FD questions the risk of malignant transformation of FD (8). This last was described as a rare complication, affecting 2,5-2,8% of adults, especially after 30-40 years (47–49). Atypical radiographic features such as cortical destruction and extension of the lesion into the adjacent soft tissue are suggestive of malignancy (48). Malignant transformation should also be considered if a patient presents a rapidly expanding lesion associated with new focal pain (49). The risk of malignancy is increased in case of prior irradiation therapy (48), polyostotic form of FD, MAS, or Mazabraud syndrome (47). Cases of locally aggressive FD are also reported, most affecting the maxillary or the mandibular region in youngs patients (50–52). To the best of our knowledge, no case as severe as the one of our patient was reported (8). Finally, we reported another rare complication with the case of Chiari I malformation. FD is associated to increased prevalence of both Chiari I malformation and basilar invagination, occurring in 6,3% and 7,6% of subjects respectively, similar in prevalence to that seen in other metabolic bone disorders, such as osteogenesis imperfecta (53–57).

### 4.7 Hepatobiliary manifestations

In our cohort, we described two cases of neonatal hepatic dysfunction including one case secondary to a syndrome to Cushing syndrome and the other case secondary to giant cell hepatitis. This last has been reported in the other cases, but the underlying mechanism is unclear (58). Although hepatobiliary dysfunction appears to be a rare feature of MAS, neonatal cholestasis and hepatitis may therefore be the first manifestation of the syndrome (58, 59). The underlying mechanism by which the constitutive activity of the G protein leads to cholestasis is unclear, however it has been suggested to play a role in bile metabolism (60). Existing descriptions of cholestasis in MAS suggest a benign phenotype with stabilization or resolution over time (60). However, a severe presentation of neonatal cholestasis leading to liver transplantation has been reported (59). Another rare hepatic manifestation found in a patient of our cohort is a hepatocellular adenoma. This last has already been described in adults but this is the first report in children (61, 62). The association of MAS and hepatocellular adenoma could potentially be explained by STAT3 The association of MAS and hepatocellular adenoma could potentially be explained by STAT3 (inflammatory pathway) activation induced by GNAS activation activation induced by *GNAS* activation (63). Other liver and gastrointestinal tract abnormalities have been described in adults including: intraductal papillary mucinous neoplasms (61, 62, 64), hemangiomas (61), gastric polyps (64), biliary cysts (61). The rate of radiographic abnormalities reaches 32% to 56% (61, 62). Thus, MAS may include a broad spectrum of liver and gastrointestinal tract abnormalities.

### 4.8 Infantile glaucoma

To the best of our knowledge, we described the first case of infantile glaucoma in MAS. Our patient presented a particularly severe form of MAS with several affected organs (bones, thyroid, ovaries, adrenals, skin). Her clinical presentation included encephalopathy of uncertain etiology. The broad extent of her phenotype with involvement of multiple tissues could explain why no other case has been described so far. Since the classic manifestations of MAS are not associated with glaucoma, the latter may be a direct consequence of MAS. However, the underlying mechanisms are unclear.

## 5 Conclusion

MAS is a multisystemic disorder, with a variable combination of symptoms, and a broad range of severity. This retrospective study conducted on a cohort of children provides a description of the clinical spectrum in MAS. It emphasizes the extent of the phenotypes patients can present. We focused on unusual complications and reported some original and rare manifestations. These uncommon abnormalities mostly occurred in patients with significant involvement of multiple other tissues. Understanding the diverse features of this rare disease is important to detect potentially significant complications. As a complement to our study, it would be interesting to carry out a study exploring FD/MAS patient’s perceptions about their disease and its impact on their quality of life, as we have recently studied in adults (65).

## Data Availability

The original contributions presented in the study are included in the article/supplementary material. Further inquiries can be directed to the corresponding authors.
